# Arctic Lineage-Canine Distemper Virus as a Cause of Death in Apennine Wolves (*Canis lupus*) in Italy

**DOI:** 10.1371/journal.pone.0082356

**Published:** 2014-01-20

**Authors:** Daria Di Sabatino, Alessio Lorusso, Cristina E. Di Francesco, Leonardo Gentile, Vincenza Di Pirro, Anna Lucia Bellacicco, Armando Giovannini, Gabriella Di Francesco, Giuseppe Marruchella, Fulvio Marsilio, Giovanni Savini

**Affiliations:** 1 Istituto Zooprofilattico Sperimentale dell'Abruzzo e del Molise “G. Caporale”, Teramo, Italy; 2 Unit of Infectious Diseases, Faculty of Veterinary Medicine of Teramo, Teramo, Italy; 3 Veterinary Services, National Park of Abruzzi, Lazio and Molise, Pescasseroli (AQ), Italy; Fordham University, United States of America

## Abstract

Canine distemper virus (CDV) infection is a primary threat affecting a wide number of carnivore species, including wild animals. In January 2013, two carcasses of Apennine wolves (*Canis lupus*) were collected in Ortona dei Marsi (L'Aquila province, Italy) by the local Veterinary Services. CDV was immediately identified either by RT-PCR or immunohistochemistry in lung and central nervous tissue samples. At the same time, severe clinical signs consistent with CDV infection were identified and taped (**Videos S1–S3**) from three wolves rescued in the areas surrounding the National Parks of the Abruzzi region by the Veterinary Services. The samples collected from these symptomatic animals also turned out CDV positive by RT-PCR. So far, 30 carcasses of wolves were screened and CDV was detected in 20 of them. The sequencing of the haemagglutinin gene and subsequent phylogenetic analysis demonstrated that the identified virus belonged to the CDV Arctic lineage. Strains belonging to this lineage are known to circulate in Italy and in Eastern Europe amongst domestic dogs. To the best of our knowledge this is the first report of CDV Arctic lineage epidemics in the wild population in Europe.

## Introduction

Canine distemper virus (CDV) belongs to the genus *Morbillivirus* of the *Paramyxoviridae* family and causes distemper. Distemper is a highly contagious and severe systemic disease frequently involving the respiratory, gastrointestinal, and nervous systems [1]–[3]. The host spectrum of CDV is broad and includes wild and domestic carnivores as well as marine mammals [4]–[7]. CDV has a non-segmented single stranded negative RNA genome of nearly 16 kb encoding six viral proteins. One of these is the haemagglutinin protein (H) coded by the H gene. H protein is a major determinant for virus-host interaction and viral entry as its binding site and binding pocket recognize signalling lymphocyte activation molecule (SLAM)-containing cell surface receptors on host [8]. The H protein appears to control the host range and tropism as well as eliciting a protective immune response [9], [10]. The importance of the H gene in the context of the life cycle of the virus has been further highlighted by the occurrence of high genetic variation within the strains detected worldwide [11]–[16]. A cluster or lineage classification has been proposed based upon the genetic relatedness of the H gene of the currently circulating CDVs. Seven clusters have been identified so far including America-1, America-2, Arctic-like, Asia-1, Asia-2, Europe and Europe Wildlife [17], [18]. Recently, a putative Asia-3 lineage has been proposed [16]. In addition, CDV strains identified in Africa, Argentina and Mexico appear to diverge from the other clusters and might represent separate geographic groups [19]–[21]. During the last three decades CDV has been detected in free-living and captive wild animals, thus CDV may represent an important conservation threat.

In this report we describe the clinical outcome and the molecular characterization of an unexpected CDV outbreak within the population of Apennine wolves living in Central Italy, mainly in the Abruzzi Region. Abruzzi contains significant protected natural habitats including the National Park of Abruzzi, Lazio and Molise, the Gran Sasso, the Monti della Laga, and the Majella National Parks. These areas host some big predators such as the Apennine wolf (*Canis lupus*) and the Marsican brown bear (*Ursus arctos marsicanus*) [22], the latter a is a threatened small and isolated population [23] of the European brown bear (*Ursus arctos*, Linnaeus, 1758), with an estimated population of about 40 individuals (95% CI: 37–52) [24].

### Ethics statement

The live wolves rescued were handled in strict accordance with the Italian Ministry for Environment, Territory and Sea recommendations, and all animal care was approved by the Veterinary Services of the National Park of Abruzzi, Lazio and Molise (PNALM). The Veterinary Services of PNALM approved entirely this study including the sampling of the animals. Post-mortem examinations and sampling were performed in the facilities of the Istituto Zooprofilattico Sperimentale dell'Abruzzo e Molise “G. Caporale” (IZSAM), Teramo-Italy, accordingly to the Ministry of Health recommendations.

## Materials and Methods

**Figure 1:**
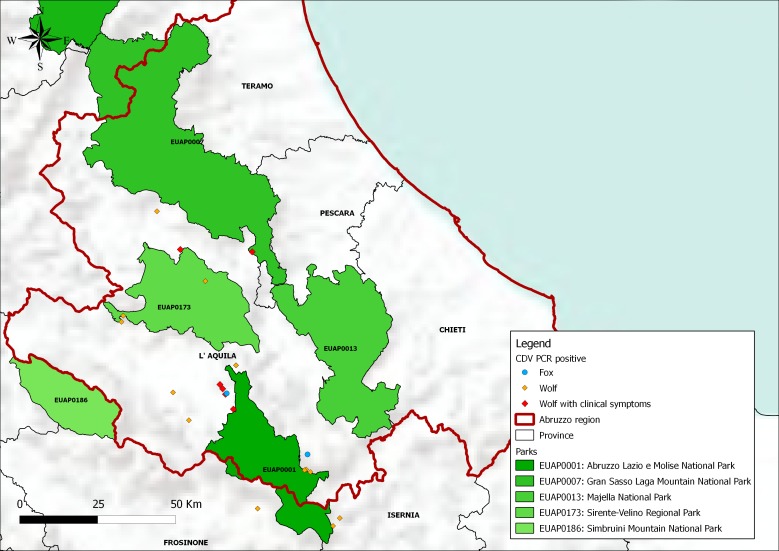
Map illustrating the distribution of CDV positive animals in Abruzzi region. Map was generated using Quantum GIS (QGIS) software, version 1.8.0.

On January 5^th^ 2013, two carcasses (Wa and Wb) of Apennine wolves (*Canis lupus*) of <1 year of age were found dead by the local Veterinary Services in an area surrounding the municipality of Ortona dei Marsi (province of L'Aquila), near the borders of the PNALM (Fig. 1). The carcasses were in an advanced state of decomposition and were sent to the laboratories of the IZSAM for the official diagnosis. The lung samples were stored in RNAlater RNA Stabilization Reagent (Qiagen S.p.A., Milan, Italy). Immunohistochemistry (IHC) was carried out on central nervous tissue and lung tissue sections using a mouse monoclonal antibody anti-CDV nucleoprotein, at the final dilution of 1∶20. Tissue sections were previously heat-treated for antigen retrieval (121°C for 8 min in citrate buffer 0.01M pH 6.0) and immune reactions were revealed using a peroxidase technique (Envision Plus kit, Dako). Positive and negative controls were included in all IHC reactions. Diagnostic RT-PCR reaction was performed for detection of CDV RNA [25]. From January 30^th^ to February 1^st^ 2013, the Veterinary Services of the PNALM observed marked clinical signs consistent with CDV infection in two other Apennine wolves (W1 and W2, of <1 year and of nearly 2 years of age, respectively) in the area surrounding the municipality of Gioia dei Marsi (L'Aquila province) (Fig. 1), 19 km distant from Ortona dei Marsi. The two wolves were rescued. A third wolf (W3) of <1 year of age, was also rescued on February 16^th^ 2013 in the area of Gioia dei Marsi. The rescued wolves were kept in isolation in the veterinary holdings of the park for first care. Ocular swabs, gastric secretions, whole blood and serum samples were taken and sent to the laboratories of the Faculty of Veterinary Medicine of Teramo for further investigations. Indirect immunofluorescence (IIF) [26] for IgM was performed on serum samples of W1, W2 and W3 whereas specific CDV neutralizing antibodies were detected by serum-neutralization (SN). During the month of February 2013, three additional wolves (W4, W5 and W6) showing clinical signs, were rescued by the Veterinary Services, these wolves succumbed immediately prior to hospitalization. Thereafter, and till April 17^th^ 2013, a total number of 30 carcasses of wolves were sent to the IZSAM. PCR (Genekam Biotechnology, Germany) for canine parvovirus (CPV-2) has been performed using DNA extracted from the intestinal contents of the wolves. As for amplification of the H encoding gene, RNA was extracted from the ocular swab samples of W1 and W3 by Trizol and Phenol/Chlorophorm method. Conversely, from the infected lung samples of Wa and Wb were stored in RNAlater, the RNA was extracted by using the QIAamp® RNeasy Mini Kit (Qiagen). From the extracted RNAs, partial length (968 bp) and full-length (1824 bp) H gene sequences were amplified by RT-PCR following procedures described in Di Francesco *et al.* [27] and Mochizuki *et al.* [11], respectively. DNA samples generated from two different RT-PCR runs were sequenced. Amplicons were used for direct sequencing in both directions using internal primers. Raw sequence data were assembled and translated into amino-acid sequences in order to check the presence of stop codons in the sequence (DNAstar, Madison, WI). Consensus sequences were aligned with H gene sequences available in GenBank and elaborated with ClustalW available in MEGA [28] version 4. Sequence alignment is available in SM, Alignment S1. Phylogenetic analysis was performed using MEGA version 4 and the evolutionary distance was computed using the maximum composite likelihood algorithm [29]. Accession numbers of sequences retrieved for the analysis are available in SM, Table S1. Statistical support was provided by bootstrapping over 1000 replicates and bootstrap values >70% were indicated at the corresponding node. The presence of potential glycosylation sites (N-X-S/T) in the H proteins was investigated with the NetNglyc server (http://www.cbs.dtu.dk/services/NetNGlyc/).

## Results

**Figure 2:**
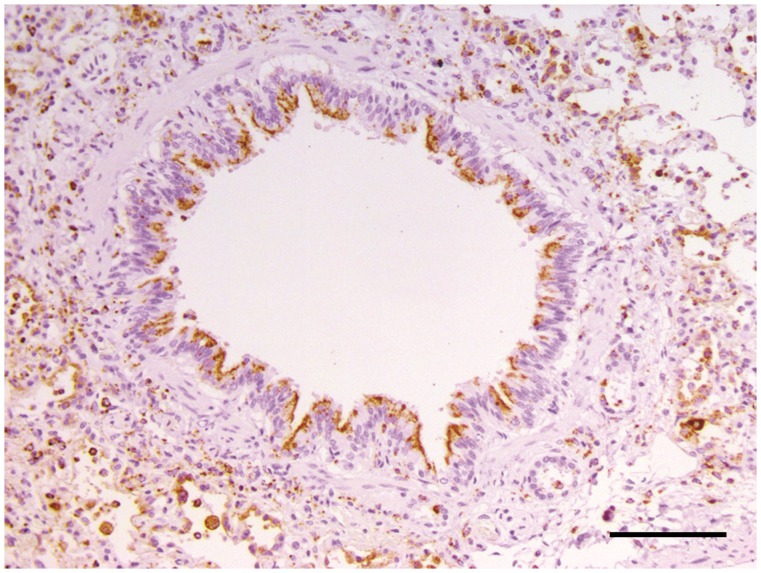
Immunohistochemistry (IHC) for CDV on lung tissue of wolf Wa. Bronchiolar epithelial cells and inflammatory cells filling the airways show a strong and specific CDV immuno-reactivity. Scale bar = 100 µm.

**Figure 3:**
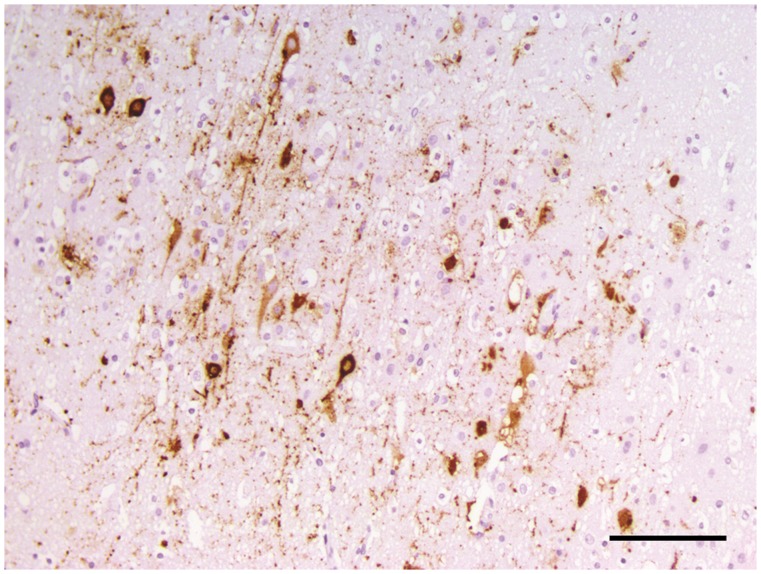
Immunohistochemistry (IHC) for CDV on cerebral cortex tissue of wolf Wa. xNeurons from the cerebral cortex show a strong and specific CDV immuno-reactivity. Scale bar = 100 µm.

At necropsy, poisoning was suspected at first glance. CDV infection was then considered after completion of necropsy and histological examination of the carcasses of Wa and Wb. Microscopically, diffuse interstitial pneumonia and characteristic syncytial giant cells were observed in Wa and Wb lung samples. CDV infection was confirmed in wolves by IHC (Fig. 2 and 3) and RT-PCR for CDV detection; both tests were performed on lung and central nervous tissues.

Clinical signs from rescued wolves were taped. Lethargy, ataxia, body temperature of 37.5°C, hemorrhagic diarrhea, muco-purulent nasal and ocular discharge, hyper-salivation and chewing movements of the jaw were observed in W1 (Video S1) whereas lethargy, ataxia, body temperature of 39°C, myoclonus, involuntary twitching of muscles, serous ocular discharge were observed in W2 (Video S2). W3 showed lethargy, ataxia, body temperature of 34°C, muco-purulent nasal discharge, muco-purulent ocular discharge, and circling (Video S3). Therapy for all of the wolves consisted of administration of fluids, cortisone and antibiotics; however, they succumbed within few days.

**Figure 4:**
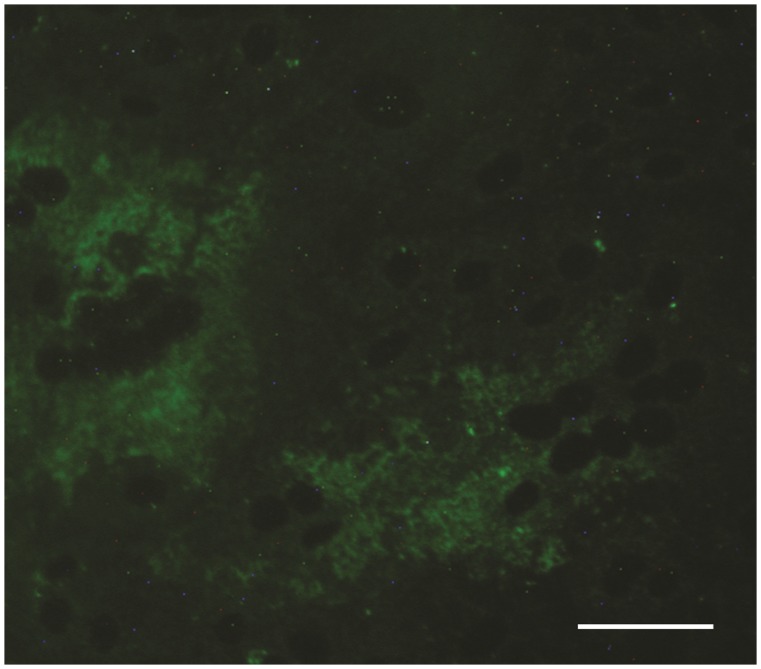
Indirect immunofluorescence (IIF) for detection IgM antibodies performed on the serum sample of wolf W1. Scale bar = 100 µm.

IIF for IgM was performed on serum samples confirming the presence of CDV specific IgM antibodies in all animals (Fig. 4) whereas SN showed specific neutralizing CDV titers of 1∶20 in W1 and W2 and 1∶10 in W3, respectively. The carcasses of the six wolves rescued (W1–W6) and the ocular swabs from W1 and the gastric secretions from W3 turned out positive for CDV either by RT- PCR or IHC. Of the 30 wolves sent to the IZSAM, 20 were found CDV positive either by IHC or RT-PCR; interestingly, five were positive for CPV-2 by PCR (Table 1). The remaining 10 wolves in which CDV was not detected died because of non-infectious causes including poisoning, traumas, poaching (data not shown).

**Table 1:** Data on wild animals infected with CDV and detected by RT-PCR.

Data Prelievo	ID	Specie Campione	Clinical symptoms	Age	CDV	CPV-2	Municipality	Province
28/08/2012	B9547PE	BADGER	yes	<1 year	IHC		VILLAMAGNA	CHIETI
01/10/2012	B11907PE	BADGER	yes	Adult	IHC		LAMA DEI PELIGNI	CHIETI
31/12/2012	W71AZ	WOLF	no	Elderly	+		COLLELONGO	L' AQUILA
05/01/2013	W63AZF	Wa	no	<1 year	+		ORTONA DEI MARSI	L' AQUILA
05/01/2013	W63AZM	Wb	no	<1 year	+	+	ORTONA DEI MARSI	L' AQUILA
08/01/2013	W196AZ	WOLF	no	Adult	+	+	SETTEFRATI	FROSINONE
12/01/2013	W601AZ	WOLF	no	<1 year	+	+	L'AQUILA	L' AQUILA
30/01/2013	W1	WOLF	yes	<1 year	+		GIOIA DEI MARSI	L' AQUILA
01/02/2013	W2	WOLF	yes	Sub-adult	+		GIOIA DEI MARSI	L' AQUILA
03/02/2013	W6	WOLF	yes	<1 year	+		SAN DEMETRIO NE' VESTINI	L' AQUILA
13/02/2013	W782AZ	WOLF	no	Adult	+		TRASACCO	L' AQUILA
15/02/2013	W4014TE	WOLF	no	Adult	+		CIVITELLA ALFEDENA	L' AQUILA
16/02/2013	W3	WOLF	yes	<1 year	+		GIOIA VECCHIA	L' AQUILA
18/02/2013	W4	WOLF	yes	Adult	+		CAPESTRANO	L' AQUILA
18/02/2013	W5	WOLF	yes	<1 year	+		GIOIA DEI MARSI	L' AQUILA
22/02/2013	W982AZ	WOLF	no	Sub-adult	+	+	MASSA D'ALBE	L' AQUILA
22/02/2013	F980AZ	FOX	no	Adult	+		GIOIA DEI MARSI	L' AQUILA
23/02/2013	W1008AZ	WOLF	no	<1 year	+		BARREA	L' AQUILA
07/03/2013	W5448TE	WOLF	no	<1 year	+		TIONE DEGLI ABRUZZI	L' AQUILA
12/03/2013	W1400AZ	WOLF	no	<1 year	+		CIVITELLA ALFEDENA	L' AQUILA
13/03/2013	F1401AZ	FOX	no	Adult	+		PESCASSEROLI	L' AQUILA
23/03/2013	W1654AZ	WOLF	no	Adult	+		CASTEL SAN VINCENZO	ISERNIA
24/03/2013	W1646AZ	WOLF	no	Adult	+		CASTEL SAN VINCENZO	ISERNIA
24/03/2013	W1647AZ	WOLF	no	Sub-adult	+	+	MASSA D'ALBE	L' AQUILA

CDV, canine distemper virus; CPV-2, canine parvovirus type 2. IHC, immunohystochemistry.

**Figure 5:**
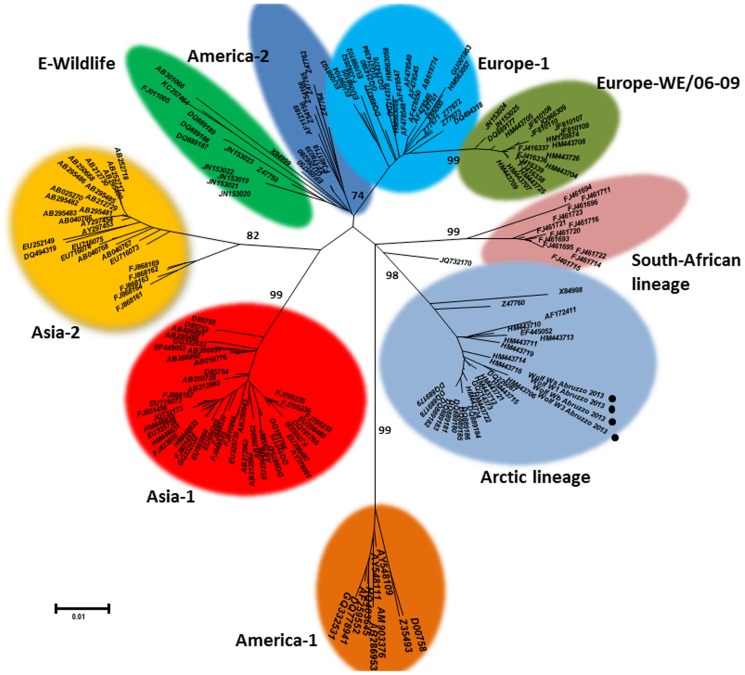
Neighbour-joining tree inferred from multiple nucleotide sequence alignment of the H encoding gene of selected CDV strains retrieved from GenBank. Sequences obtained in this study are marked with a circle.

The nucleotide sequences were deposited in GenBank (Wa-CDV2013, KC966928; Wb-CDV2013, KC966929; W1-CDV2013, KC992186; W2-CDV2013, KC992187); the sequences were found to share 100% nucleotide identity between them. Phylogenetic analysis conducted using either partial (W1 and W3) or full-length (Wa-CDV2013 and Wb-CDV2013) H gene sequences revealed that the strains involved in the distemper outbreak of the Apennine wolves cluster together in the Arctic lineage (Fig. 5). They bear the highest nucleotide identity with CDV strains Dog RO_11_08 (99.3%), 48/05 (99%) and 179/94 (98.9%) isolated from dogs in Italy. The inferred amino acid sequences of the H protein Wa-2013 and Wb-2013 (607 aa) showed a Y residue at position 549. A total of eight potential glycosylation sites (N-X-S/T) were recognized at positions 19–21, 149–151, 309–311, 391–393, 422–424, 456–458, 587–589, and 603–605. Whereas all these glycosylation sites are conserved in CDVs among the major European lineage, site 309–311 is missing in CDV strains of lineage America-1 (including the vaccine strains) and the glycosylation site 603–605 is missing in the vaccine strain Onderstepoort. Unfortunately, Wa-CDV2013 and Wb-CDV2013 could not be propagated in tissue culture thus hampering further analysis of viral mRNAs in the infected cells.

## Discussion

The present study showed that a CDV strain belonging to the Arctic lineage infected and killed 20 Apennine wolves of different ages in the Abruzzi region during the winter of 2013. Previous studies demonstrated that at least three distinct CDV lineages are circulating in Italy including Europe-1, Europe Wildlife and Arctic lineages [13], [17], [27], [30], [31]. Europe-1 lineage is commonly found in domestic dogs all over the European countries. The Europe Wildlife lineage strains are detected in wild animals (fox, mink) and domestic dogs in Hungary [31]. Unlike Europe-1 and Europe Wildlife, CDV strains of the Arctic lineage are detected only in domestic dogs in Italy, Hungary and North America. They are called Arctic because they were first identified in the susceptible population of the Arctic ecosystem. Only recently, a CDV Arctic strain was identified in one breeding fox in China. Details on the clinical outcome and distribution, however, remain unknown [16].

To the best of our knowledge, this is the first case of a CDV Arctic strain infecting wolves, only infections in domestic dogs have been previously described. Sequence analysis revealed a high genetic relatedness between the wolf strains and the recent Arctic strains detected in Italian dogs, including the presence of Y549 and eight potential N-Glycosylation sites in the H protein [13]. However, considering that we sequenced the H encoding gene from the RNA purified from the infected tissues of only four of the 20 infected wolves, the co-circulation of CDV strains belonging to other lineages cannot be excluded. Overall, the finding of CDV of the Arctic lineage in wolves certainly represents a novelty and raises several questions regarding its origin and spreading. A survey conducted between 2006 and 2009 by Monne and colleagues [13] revealed the co-circulation in the same area (Northeastern Regions of Italy) of two distinct CDV lineages: cluster WE/06-09 of the Europe-1 lineage in badgers and foxes, and the Arctic lineage strains in dogs without any spillover in both directions. The authors speculated that this phenomenon may depend on either a lack of contact between the two populations or the strong adaptation of the WE/06-09 to the wild hosts. This latter assumption is supported by the presence of Y549H mutation in the H protein of the CDV strains isolated from badgers and foxes and the preservation of Y549 in the Arctic strains of dogs. Indeed, Y549H mutation occurs predominantly in CDV isolates from non-dog host species [18]. Likewise, the presence of Y549 in the H protein of Wa-CDV2013 and Wb-CDV2013 suggests the domestic dog as the virus reservoir for wolves this would exclude the presence of a CDV strain adapted to the local wild population. Accordingly, direct contact between wolves (or other wildlife animals) and CDV infected dogs could be the starting event of the current epizootics.

In the Abruzzi region, wolves are often seen near villages, urban roads and farms. In addition, transhumance of sheep with shepherd dogs is still practiced in proximity of places with wildlife. During the CDV epizootic in wolves, many cases of clinical distemper in unvaccinated domestic, feral and shepherd dogs were also reported by the veterinary local authorities suggesting that this strain was likely circulating in naïve dogs prior to the detection of CDV in the wildlife population. Alternatively spillover in domestic dogs from urban wildlife could also be possible scenario [32].

Two red foxes (*Vulpes vulpes*) and two badgers (*Meles meles*) from the same area also died because of distemper infection. CDV was detected either by RT-PCR or IHC. Unfortunately, it was not possible to perform any molecular analysis from the infected tissues because of the scarce amount of sample. The CDV detected in the foxes is likely to be the same as the CDV of the wolves considering the timing and their geographical recovery municipalities. The two badgers were rescued in the Province of Chieti, municipalities of Villamagna and Lama dei Peligni, respectively (both distant 90 km from Ortona dei Marsi) in the summer/autumn of 2012. These two events may suggest a multi-host epizootic, in which wolves likely played a major role in the CDV geographical amplification considering the wide geographic range over which juvenile individuals migrate during the period of dispersal. As is often the case in extremely contagious viruses like CDV, the rise in host concentration may have enhanced the efficacy of virus transmission.

Although the high genetic variation of the H gene raises questions regarding the efficacy of the CDV strains currently used for the attenuated vaccines production [17], vaccination remains the basic preventive measure for dogs. It has been shown to be highly efficacious and long lasting if properly administered. Domestic dogs need to be vaccinated and protected. Vaccinations enable a high immunity in the population hence controlling CDV infection, therefore only the occurrence of sporadic cases may be observed. Thus, in order to prevent future and potential devastating distemper outbreaks in the wild population, vaccination strategies should be implemented particularly within the dogs living in rural areas and captured dogs housed in local kennels.

Distemper represents a major conservation threat and has contributed to the decline of several wild terrestrial and aquatic mammals [5], [33]–[35]. Regional and National parks cover more than 1/3 of the entire extension of the Abruzzi region and harbor several animal species including Apennine wolf, Marsican brown bear, red fox, wild cat (*Felid silvestris*), stone marten (*Martes foina*), badger, European pine marten (*Martes martes*), European polecat (*Mustela putorius*), and least weasel (*Mustela nivalis*), all susceptible to CDV infection [4], [36]. In general available data regarding CDV outbreaks in the European wild carnivore populations are limited to a few reports from Germany, Spain, Czech Republic and Italy [4], [27], [37]–[41]. In Abruzzi, serological investigations during catch and release programs documented CDV circulation in wolves, Marsican brown bear, red foxes and badgers (unpublished data). The main concern originating from the current distemper outbreak is the chance that CDV may affect the population of the Marsican brown bear. CDV was previously shown to circulate in the Marsican brown bear of the park [36], approximately 40 (95% CI: 37–52) [24] bears live in the PNALM. However, at that time no clinical signs were observed in the bears.

A recent survey suggests that Arctic CDV strains did not circulate in the Abruzzi region before 2009 [27]. As the Arctic lineage CDV represents a new introduction into a naïve ecosystem, we cannot rule out that mutations may occur in the viral genome thus increasing the chances of expanding its host range to include the bear and cause clinical disease [42]. The fight against the uncontrolled trading of low cost pets from Eastern Europe, implementation of surveillance activities and vaccination strategies, and further studies regarding antigenic mapping of CDV strains circulating in the susceptible population are warranted in order to prevent the loss of the endangered wild species.

### Genbank accession numbers

H gene of Wa-CDV2013, KC966928

H gene of Wb-CDV2013, KC966929

H gene of W1-CDV2013 KC992186

H gene of W3-CDV2013 KC992187

## Supporting Information

Video S1 **Clinical signs observed in wolves W1.**
(MP4)

Video S2 **Clinical signs observed in wolves W2.**
(MP4)

Video S3 **Clinical signs observed in wolves W3.**
(MP4)

Alignment S1 **Alignment of the haemagglutinin gene nucleotide sequences from selected CDV strains available in Genbank.**
(TXT)

Table S1 **Genbank accession numbers of CDV strains used for the phylogenetic analysis.**
(XLSX)
